# Gkongensin A, an HSP90β inhibitor, improves hyperlipidemia, hepatic steatosis, and insulin resistance

**DOI:** 10.1016/j.heliyon.2024.e29367

**Published:** 2024-04-09

**Authors:** Kun Miao, Yawei Zhao, Ning Xue

**Affiliations:** a Department of Hand Surgery, Fuzhou Second General Hospital, 350007, Fuzhou, Fujian, China; b Department of Pharmacy, Jurong Hospital Affiliated to Jiangsu University, Jurong, 212400, Jiangsu, China; c Department of Acupuncture, Jurong Hospital Affiliated to Jiangsu University, Jurong, 212400, Jiangsu, China

**Keywords:** Kongensin a, Hsp90β, SREBPs, DNL, Hyperlipidemia, Insulin resistance

## Abstract

The prevalence of obesity and its primary associated comorbidities, such as type 2 diabetes and fatty liver disease, has reached epidemic proportions, with no successful treatment available at present. Heat shock protein 90 (HSP90), a crucial chaperone, plays a key role in *de novo* lipogenesis (DNL) by stabilizing and maintaining sterol regulatory element binding protein (SREBP) activity. Kongensin A (KA), derived from *Croton kongensis*, inhibits RIP3-mediated necrosis, showing promise as an anti-necrotic and anti-inflammatory agent. It is not yet clear if KA, acting as an HSP90 inhibitor, can enhance hyperlipidemia, hepatic steatosis, and insulin resistance in obese individuals by controlling lipid metabolism. In this study, we first found that KA can potentially decrease lipid content at the cellular level. C57BL/6J mice were given a high-fat diet (HFD) and received KA and lovastatin through oral administration for 7 weeks. KA improved hyperlipidemia, fatty liver, and insulin resistance, as well as reduced body weight in diet-induced obese (DIO) mice, with no significant alteration in food intake. *In vitro*, KA suppressed DNL and reduced the amounts of mSREBPs. KA promoted mSREBP degradation via the FBW7-mediated ubiquitin-proteasome pathway. KA decreased the level of *p*-Akt Ser308, and *p*-GSK3β Ser9 by inhibiting the interaction between HSP90β and Akt. Overall, KA enhanced hyperlipidemia, hepatic steatosis, and insulin resistance by blocking SREBP activity, thereby impacting the FBW7-controlled ubiquitin-proteasome pathway.

## 1 Introduction

Excessive food intake and lack of exercise are the main factors that lead to morbid obesity, but it is very difficult to change a person's lifestyle [1,2]. Morbid obesity (class III obesity) is a chronic disease characterized by a body mass index (BMI) of 40 or above and the presence of health issues related to obesity. Morbid obesity results in significant metabolic diseases like atherosclerosis, diabetes, hypertension, and heart disease [3]. Bariatric surgery is currently the only effective option for treating morbidly obese patients [4,5]. According to government calculations, obesity is currently costing the NHS $6.1 billion and $27 billion to the broader society. New research indicates that a mere 2.5 kg reduction in weight among overweight or obese individuals could result in a savings of $105 million within 5 years [6]. To help patients with obesity and address the many complications and costs associated with obesity, it is urgently necessary to explore additional therapeutic targets and molecules [7].

Sterol regulatory element binding proteins (SREBPs), which are members of the basic helix-loop-helix-leucine zipper family of transcription factors, play a crucial role in lipid homeostasis by controlling the expression of genes related to *de novo* lipogenesis (DNL), triglyceride, cholesterol, and phospholipid synthesis [8]. A significant role played by SREBPs in lipid metabolism makes them intimately related to metabolic syndrome [8]. Humans have three acknowledged isoforms of SREBPs produced by the genes sterol-regulatory element-binding transcription factor 1 and sterol-regulatory element-binding transcription factor 2. Each of the three SREBP isoforms, SREBP-1a, SREBP-1c, and SREBP-2, has a distinct role. SREBP-1a and SREBP-1c primarily control DNL, including *ACC-1*, *SCD-1*, and *FASN*. SREBP-2 primarily enhances the expression of LDL receptors (LDLR) and enzymes related to cholesterol production, including *HMGCR*, *HMGCS*, *MVK*, *LSS,* and *FDPS* [9,10]. SREBP-1a, compared to SREBP-1c, is recognized as a stronger transcription activator due to its extended transactivation domain in the N-terminal area among the various isoforms. SREBP-1 is primarily found in the liver and pronephros, while SREBP-2 has a broader expression pattern. The endoplasmic reticulum (ER) stores SREBPs, which are inactive precursors in mammals. Activation of SREBPs occurs when insulin is present or when sterol levels are low, leading to their release from the ER [11]. Upon interacting with the SREBP cleavage-activating protein (SCAP), SREBPs are conveyed to the Golgi apparatus through COP II. At the Golgi, S1P, and S2P release the N-terminal of SREBPs (SRERPs). Afterward, nSREBPs move to the nucleus and attach to the SRE sequences of their target genes, leading to the activation of genes associated with the DNL [9,12]. Mature SREBPs (mSREBPs) are extremely unstable because they are easily degraded through ubiquitin-dependent mechanisms. GSK3 phosphorylates nSREBPs in a sterol-agnostic way, leading to ubiquitination and degradation upon interaction with FBW7, an E3 ubiquitin ligase containing F-box and WD40 domains [13]. Therefore, blocking SREBPs using small compounds may offer a new approach to treating metabolic diseases such as obesity, type 2 diabetes, and atherosclerosis [14]. Multiple research studies have shown that HSP90 supports DNL in the liver by preserving the stability and function of SREBP [15]. In their study, Zheng and his team discovered that hepatic HSP90β, rather than HSP90α, was increased in both individuals with nonalcoholic fatty liver disease (NAFLD) and obese mice. Additionally, hepatic HSP90β was associated with serum lipid levels in a clinical setting [16]. It protects Akt's Thr308 from dephosphorylation by protein phosphatase 2A (PP2A). Akt andHSP90β interact directly in the central region. Inhibiting HSP90β led to a significant reduction in Akt phosphorylation at Thr308, resulting in the inactivation of GSK3β. Consequently, GSK3β is unable to phosphorylate SREBPs, leading to the stabilization of SREBPs through the inhibition of FBW7-mediated ubiquitination. As a result, the levels of neutral lipids and cholesterol significantly dropped in cells lacking HSP90 [16]. Inhibiting SREBP activity through genetic or pharmacological suppression of HSP90β has been demonstrated to greatly improve fatty liver disease, type 2 diabetes, and atherosclerosis caused by obesity.

HSP90, a highly prevalent molecular chaperone, is essential for regulating the cell cycle, folding proteins, and transmitting signals [17]. In addition to being found in multiple tissues, HSP90 accounts for over 1 % of the total cytosolic protein. The HSP90 family mainly consists of four members: HSP75, HSP90α, HSP90β, and HSP90B1 [18]. There has been increased attention given to the progress of new genetic, biochemical, and pharmacological tools, including HSP90 explored as one of the most popular therapeutic targets in cancer and several other diseases [19]. As HSP90 inhibitors emerge, studying HSP90 has become more readily available. In addition, the scientific field is becoming increasingly interested in inhibiting specific HSP90 isoforms to study cellular signaling and pharmacology [20]. Subtle functional differentiation among HSP90 isoforms and mixed heterodimers has not yet been expounded [21].

Kongensin A (KA), a naturally occurring compound derived from *Croton kongensis*, exhibits strong inhibitory effects on HSP90β and effectively impedes RIP3-dependent necrosis, thereby demonstrating promising prospects for its application as an anti-necrotizing and anti-inflammatory agent [22]. In this study, we first reported that KA could inhibit hepatic DNL and analyzed its mechanism of action *in vitro*. Additionally, we identified the impact of KA in DIO mice, showing that KA reduced levels of lipids in the blood, inhibited lipid accumulation in the liver and white adipose tissue (WAT), enhanced insulin sensitivity, and reduced hepatic steatosis.

## 2 Materials and methods

### 2.1 Reagents

Luciferase assay reagents were purchased from Promega (Madison, Wisconsin, USA). Filipin was purchased from APExBIO (Houston, USA). 25-HC, MTT, and Nile-red were acquired from Sigma-Aldrich (St. Louis, Missouri, USA). Lovastatin was purchased from Aladdin (Los Angeles, 19 CA, USA). KA, MG-132, and Cycloheximide were purchased from MedChem Express (Shanghai, China). FBS, F12K, and DMEM medium were purchased from GIBCO (New York, USA). Protein A/G beads were purchased from Santa Cruz (Dallas, Texas, USA).

### 2.2 Cell culture

Cell culture was performed at 37 °C with 5 % CO_2_ in all cell lines, using DMEM supplemented with 10 % FBS, and HL-7702 cells were purchased from Keygen (Nanjing, China). To create HL-7702/SRE-Luc reporter cells, HL-7702 cells were transfected with pSRE-Luc, and monoclonal cell lines were eliminated by replacing the medium with hygromycin B (Roche, Germany).

### 2.3 HSP90β KO cells

CRISPR-Cas9 was used to create HL-7702 by targeting the site 5′-CACCGCCTGACAGACCCTTCGAAGT-3′, with the guidance of the CRISPR direct sequences. gRNA target sequences were created and inserted into the pHBcas9/gRNApuro plasmid, then delivered into HL-7702 cells along with HSP90β complementary oligonucleotides using *Bbs*I restriction enzyme. After being transfected for 48 h, the cells were exposed to puromycin at a concentration of 2 g/ml for 3 days.

### 2.4 Cell proliferation assay

The MTT tests were utilized to measure cell proliferation. In 96-well plates of DMEM containing 10 % FBS, 2.0 × 104 HL-7702 cells were planted for 24 h. For an additional 18 h, cells were treated with or without KA. Following a 4 h treatment with MTT (5 mg/ml) at 37 °C, 150 μl of dimethyl sulfoxide (DMSO) was introduced into each well.

### 2.5 Luciferase reporter assay

HL-7702/SRE-Luc reporter cells were placed in a 96-well plate, exposed to KA or left untreated, and cultured in DMEM with 5 % LPDS, 10 μM mevalonate, and 10 μM compactin, for 16 h. The medium was removed afterward. After being washed twice with PBS, the cells were then exposed to reporter lysis buffer for 20 min. Next, the fluid was moved to white 96-well plates, followed by the addition of the luciferase test solution.

### 2.6 Western blot analysis

HL-7702 cells were dissolved in RIPA buffer containing 50 mM Tris–HCl, pH 8.0, 1 % Nonidet P-40, 150 mM NaCl, 0.1 % SDS, 0.5 % sodium deoxycholate, and 1 mM EDTA after being washed with ice-cold PBS. PVDF membranes were blocked using PBST following SDS-PAGE separation of whole-cell extracts. Next, antibodies were attached for a prolonged period at a temperature of 4 °C. Following rinsing with PBST, a secondary antibody conjugated with HRP (Beyotime, China) was incubated for 2 h at ambient temperature. The *anti*-SREBP-2 (NB100-74543, Rabbit) antibody was from Novus Bio (Little, CO, USA). *Anti*-Myc (sc-40, mouse) and *anti*-SREBP-1 (sc-13551, mouse) antibodies were from Santa Cruz Biotechnology (Dallas, USA). The *anti*-HSP90β (ab203085, Rabbit) antibody was from Abcam (Cambridge, UK). The anti-HA-tag (AE008, mouse) antibody was from ABclonal Technology (Hubei, China). Anti-Flag-tag (14793, Rabbit) antibody was from Cell Signal Technology (Beverly, MA, USA). *Anti*-β-Actin (AF0003, mouse) antibody was from Beyotime Biotechnology (Jiangsu, China).

### 2.7 Quantitative real-time PCR

The manufacturer's recommendations were followed while using Trizol (Vazyme, Nanjing, China) for total RNA extraction from hepatocytes and liver tissue. RNA amounts were balanced and cDNA was synthesized using Hiscript II reverse transcriptase (Vazyme, Nanjing, China). Gene expression was evaluated using SYBR-green dyes and a LightCycler 96 Real-Time PCR System manufactured by Roche (Basel, Switzerland). GAPDH expression, a reference gene, served as an internal control for analyzing the data. The levels of mRNA in the liver of obese mice and hepatocytes were evaluated. Table 1 contains the primer sequences that were utilized.

**Table 1:** Nucleotide sequences of gene-specific primers used for quantitative real-time PCR, related to the experimental procedures.

Specifies	Gene name	Sequence of forward and reverse primers (5′ to 3′)
***Mus musculus***	GAPDH	TGTGTCCGTCGTGGATCTGA CCTGCTTCACCACCTTCTTGAT
	SREBP-1c	GGCCGAGATGTGCGAACT TTGTTGATGAGCTGGAGCATGT
	SREBP-2	GCGTTCTGGAGACCATGGA ACAAAGTTGCTCTGAAAACAAATCA
	SCAP	ATTTGCTCACCGTGGAGATGTT GAAGTCATCCAGGCCACTACTAATG
	HMGCS	GCCGTGAACTGGGTCGAA GCATATATAGCAATGTCTCCTGCAA
	HMGCR	CTTGTGGAATGCCTTGTGATTG AGCCGAAGCAGCACATGAT
	FDPS	ATGGAGATGGGCGAGTTCTTC CCGACCTTTCCCGTCACA
	LDLR	AGGCTGTGGGCTCCATAGG TGCGGTCCAGGGTCATCT
	ACC	TGACAGACTGATCGCAGAGAAAG TGGAGAGCCCCACACACA
	Fasn	GCTGCGGAAACTTCAGGAAAT AGAGACGTGTCACTCCTGGACTT
	SCD-1	CCGGAGACCCCTTAGATCGA TAGCCTGTAAAAGATTTCTGCAAACC
	ApoB	CGTGGGCTCCAGCATTCTA TCACCAGTCATTTCTGCCTTTG
	ApoE	GCTGGGTGCAGACGCTTT TGCCGTCAGTTCTTGTGTGACT
	GK	CCGTGATCCGGGAAGAGAA GGGAAACCTGACAGGGATGAG
	ACL	GCCAGCGGGAGCACATC CTTTGCAGGTGCCACTTCATC
	ACS	GCTGCCGACGGGATCAG TCCAGACACATTGAGCATGTCAT
***Mus musculus***	G-6-PD	GAACGCAAAGCTGAAGTGAGACT TCATTACGCTTGCACTGTTGGT
	IRS-1	GCGGGCTGACTCCAAGAAC GCTATCCGCGGCAATGG
	IRS-2	GGAGAACCCAGACCCTAAGCTACT GATGCCTTTGAGGCCTTCAC
	PEPCK	CCACAGCTGCTGCAGAACA GAAGGGTCGCATGGCAAA
	Insig-1	TCACAGTGACTGAGCTTCAGCA TCATCTTCATCACACCCAGGAC
	Insig-2a	CCCTCAATGAATGTACTGAAGGATT TGTGAAGTGAAGCAGACCAATGT
	Insig-2b	CCGGGCAGAGCTCAGGAT GAAGCAGACCAATGTTTCAATGG
	SRB1	TGGACAAATGGAACGGACTC GTGAAGCGATACGTGGGAAT
	LPL	CTTCTTGATTTACACGGAGGT ATGGCATTTCACAAACACTG
	Leptin	CGGTTCCTGTGGCTTTGG GGTCTGAGGCAGGGAGCA
	adiponectin	GTTGCAAGCTCTCCTGTTCC TCTCCAGGAGTGCCATCTCT
	PPARγ	GGCTGAGGAGAAGTCACACTCTG AAATCTTGTCTGTCACACAGTCCTG
	UCP-1	GTGAAGGTCAGAATGCAAGC AGGGCCCCCTTCATGAGGTC
	UCP-2	GCCCCTTCACCTCTTTAGCA CCAAGCACTGGGAAGGTCTAAC
***Homo sapiens***	HMGCR	TGATTGACCTTTCCAGAGCAAG CTAAAATTGCCATTCCACGAGC
	SREBP-2	AACGGTCATTCACCCAGGTC GGCTGAAGAATAGGAGTTGCC
	HMGCS-1	CTCTTGGGATGGACGGTATGC GCTCCAACTCCACCTGTAGG
	MVK	GGAGCAAGGTGATGTCACAAC CGGCAGATGGACAGGTATAAGT
	FDPS	TGTGACCGGCAAAATTGGC GCCCGTTGCAGACACTGAA
	FDFT-1	CCACCCCGAAGAGTTCTACAA TGCGACTGGTCTGATTGAGATA
***Homo sapiens***	LSS	GTACGAGCCCGGAACATTCTT CGGCGTAGCAGTAGCTCAT
	SE	CCTCTTTGTCTTTACGGTTTCC GTCCCAGTGCCTTTGATGTT
	MSMO-1	TGCTTTGGTTGTGCAGTCATT GGATGTGCATATTCAGCTTCCA
	SREBP-1c	ACAGTGACTTCCCTGGCCTAT GCATGGACGGGTACATCTTCAA
	ACC-1	ATGTCTGGCTTGCACCTAGTA CCCCAAAGCGAGTAACAAATTCT
	SCD	TCTAGCTCCTATACCACCACCA TCGTCTCCAACTTATCTCCTCC
	FASN	CCGAGACACTCGTGGGCTA CTTCAGCAGGACATTGATGCC
	SCD-2	CTCTGCGAGTGAATTTGGC GATCATCGGCTTGGTTGC

### 2.8 Animal experiments

The study's test subjects were authorized by the Science and Technology Department of Jiangsu (SYXK (SU) 2016-011) and adhered to Chinese regulations and guidelines for experimental animal management. All mice were kept under a 12 h light and 12 h dark cycle. Each mouse was given either a high-fat diet (Nantong, China) or a standard diet (Nantong, China). The HFD (TP23300) had a composition of 20.6 % carbohydrate, 60 % fat, and 19.4 % protein in terms of calories, while the XT007 normal diet consisted of 13 % fat, 60 % carbohydrate, and 27 % protein. Lovastatin is chosen as a beneficial substance, functioning as an inhibitor of 3-hydroxy-3-methylglutaryl-coenzyme A reductase (HMGCR) to decrease liver lipid synthesis and enhance hyperlipidemia. Male C57BL/6J mice, aged six weeks, were acquired from Nanjing Biomedical Research Institute in Nanjing, China, and were given a week to adjust before the start of the experiment. Next, the mice were divided into five groups randomly, each with seven members: a group treated with vehicle and chow, a group treated with vehicle, a group treated with lovastatin (30 mg/kg/day), and a group treated with KA (3 or 6 mg/kg/day). DIO mice received oral administration of lovastatin or KA diluted in 0.5 % CMC-Na for a period of 7 weeks. Animal experiments were conducted following the authorized protocols.

### 2.9 Tests for glucose and insulin tolerance

Mice that had been fasting for the previous night and had unrestricted access to water underwent glucose tolerance tests (GTT) and insulin tolerance tests (ITT). Mice were administered either 2 g/kg of glucose (Sigma) via gavage or 0.75 U/kg of insulin (Sigma) intravenously. Tail blood glucose levels were assessed 15, 30, 60, or 120 min after the injection. After three days of the glucose or insulin tolerance tests, the animals were euthanized by cervical dislocation, and samples of blood, intercapsular brown adipose tissue, epididymal white adipose tissue, and liver were collected. The calculation of the area under the curve (AUC) was used to measure the results of the GTT and ITT.

### 2.10 Serum lipid, glucose, insulin, and aminotransferase detection

Biochemical analysis was conducted on alanine aminotransferase (ALT), aspartate aminotransferase (AST), total cholesterol (TC), triglycerides (TG), low-density lipoprotein cholesterol (LDL-c), high-density lipoprotein cholesterol (HDL-c), and glucose using Biotek biochemistry analyzers from Vermont, USA, and Nanjing Jiancheng Bioengineering Institute from Nanjing, China. The levels of serum insulin were measured using ELISA kits from CUSABIO, Wuhan, China.

### 2.11 Examination of liver and fat tissues at a microscopic level

The white adipose tissues (WAT), brown adipose tissues (BAT), and livers were fixed at 4 °C in 4 % paraformaldehyde. Embedded in paraffin wax overnight, the paraffin sections are cut at 5 μm and stained with hematoxylin and eosin. Liver samples are placed in Tissue-Tek OCT cryostat molds from Leica Biosystem in Shanghai, China, and then stored at −20 °C for freezing. Following this, the samples were sliced at 10 m using a cryostat and then treated with 0.5 % oil red O from Sigma in the United States.

### 2.12 Co-immunoprecipitation

To conduct co-immunoprecipitation assays, 293T cells were transfected with different plasmids (Myc–HSP90β, Flag-mSREBP-1c, HA-ubiquitin, and HA-Akt) and incubated for 24 h. After washing the cells three times at 4 °C, they were lysed using a buffer containing 20 mM Tris-HCl, pH 7.5, 137 mM NaCl, 5 mM EDTA, 1 % NP-40, 10 % glycerol, 50 mM NaF, 1 mM Na_3_VO_4_, and PMSF protease inhibitor. After centrifuging the cell lysate at 12,000 rpm for 10 min at 4 °C, 10 % of the supernatant was utilized for Western blot analysis, while the rest was left to incubate with specified antibodies overnight at 4 °C. Furthermore, Santa Cruz protein A/G plus agarose beads were introduced at 4 °C for an additional 2 h. Subsequently, five washes with cold PBS were performed before proceeding to western blotting.

### 2.13 Cholesterol and TG measurement

To quantify intracellular TC and TG levels, cells were grown in 6-well dishes and then resuspended in 1 ml of PBS. About 100 μl of the complete mixture was moved to a fresh tube and spun at 1000 times the force of gravity for 5 min at 4 °C. Following cell lysis in lysis buffer, a fraction of the mixture was utilized for measuring protein levels while the remainder was dedicated to extracting lipids. Cells that were gathered underwent centrifugation at 1000*g* in water for 5 min at 4 °C, then combined with 1 ml of 0.1 M sodium chloride and 1 ml of chloroform-methanol (2:1, v/v) for 3 h at 24 °C. After that, they were centrifuged at 3700 rpm for 10 min. Dryness was achieved by transferring and evaporating the lower organic phase. A kit for determining TC or TG concentrations, manufactured in Shanghai, China, was utilized to measure the levels of remaining liquid in 50 μl of 1 % Triton-X 100 dissolved in absolute ethanol. A sample of liver tissue was homogenized in PBS for 0.5 ml until about 40–50 mg of TC and TG were determined. Approximately 5 μl of the entire homogenates were required for protein analysis.

### 2.14 Statistical methods

Prism software (GraphPad Prism 8, USA) was used to analyze all the data. One-way ANOVA with Dunnett's *post hoc* test was used to analyze multiple groups. Differences with p < 0.05 were considered statistically significant.

## 3 Results

### 3.1 KA decreases cellular lipid levels

**Fig. 1:**
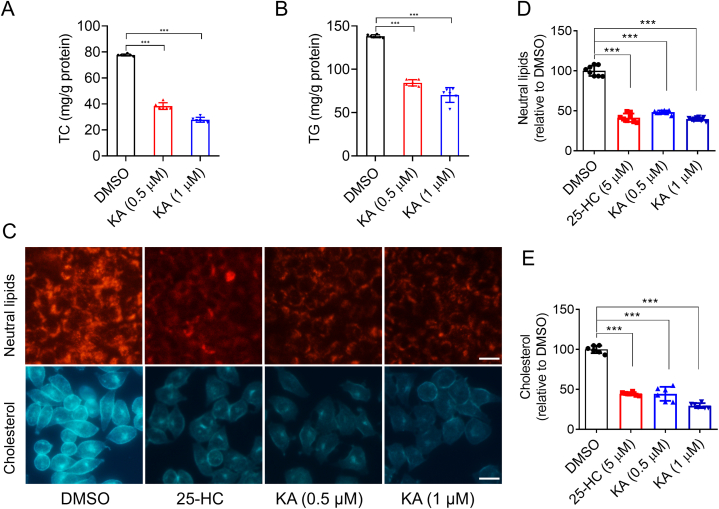
KA decreases the level of lipids in hepatocytes. (A–B) The cellular TC (A) and TG (B) levels were measured in human liver HL-7702 hepatocytes treated KA with 0.5 or 1 μM for 24 h. Statistical analysis was done with one-way ANOVA (Dunnett's post hoc test). (C) The HL-7702 hepatocytes were treated KA with 0.5 or 1 μM for 24 h. After that, the cells then were stained with Nile-Red, which specifically recognizes neutral lipids or filipin, which definitely binds free cholesterol. (D–E) Statistical analysis of the results in Fig. 1C. Statistical analysis was done with one-way ANOVA (Dunnett's *post hoc* test). *p < 0.05, **p < 0.01, ***p < 0.001 *vs* DMSO.

KA has been identified as an antitumor, antimetastatic, and antiangiogenic ingredient. Nevertheless, there have been no reports on the impact of KA on the accumulation of liver fat and insulin resistance. Here, we established an *in vitro* assay system to screen low-lipid compounds from a natural product library. In a dose-dependent manner, it was discovered that KA notably decreased the concentrations of TC and TG in HL-7702 cells (Fig. 1A and B). Additionally, Nile-red and filipin staining indicated that KA reduced lipid droplets and cellular cholesterol levels in a dose-dependent manner (Fig. 1C–E).

### 3.2 KA protects against weight gain and lipid abnormalities in DIO mice

**Fig. 2:**
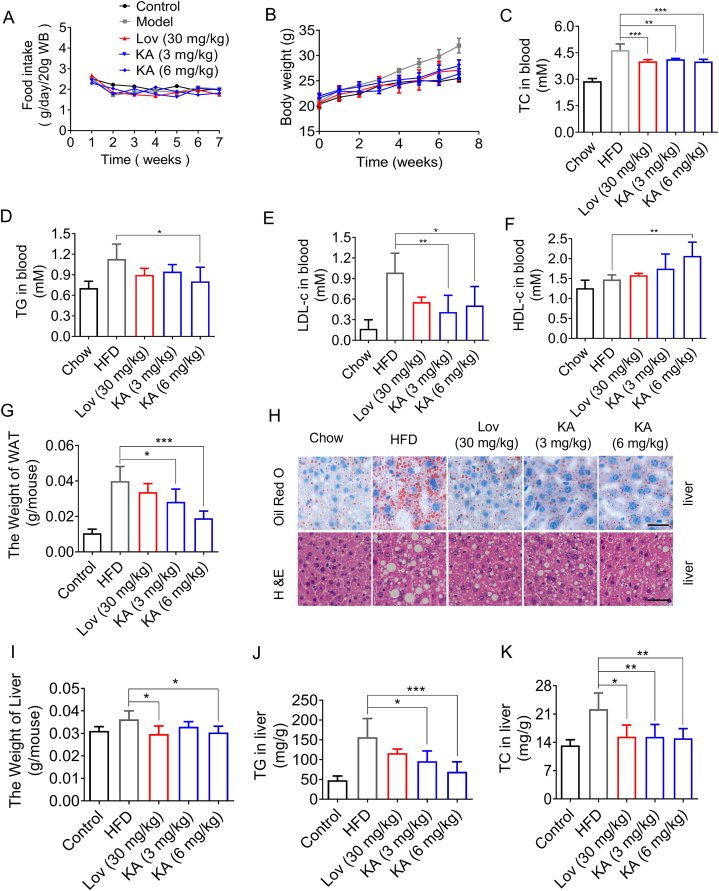
KA improves the lipid metabolism disorder in DIO mice. (A) Body weight and (B) food intake. (C–F) The TC (C), TG (D), LDL-c (E), and HDL-c (F) levels in the blood of each group of mice. (G) The ratio of fat and body weight. (H) Representative gross morphology and oil red staining of the mouse livers, and histological analysis of liver, WAT, and BAT. (I) The weight of the liver. (J and K) The effect of KA on TG (J) and TC (K) in the liver. Statistical analysis was done with one-way ANOVA (Dunnett's *post hoc* test). *p < 0.05, **p < 0.01, ***p < 0.001 *vs* Model.

In order to confirm the impact of KA in living organisms, C57BL/6J mice fed a high-fat diet were administered either vehicle (0.5 % CMC-Na, chow), lovastatin (an HMGCR inhibitor), or KA (30 or 60 mg/kg/day) for a duration of 7 weeks. Throughout the procedure, there were no notable variations in food consumption among any of the groups (Fig. 2A). Similar to lovastatin-treated obese mice, KA treatment significantly lowered obesity-induced body weight gain (Fig. 2A and B). Subsequently, lipid concentrations were measured in the blood, liver, and fat tissues to assess the possible impact of KA on reducing lipid accumulation in DIO mice. As shown in Fig. 2C and D, in mice treated with lovastatin and KA, the serum levels of TC and TG were notably reduced compared to DIO mice. KA had a comparable effect on HDL-c and LDL-c as lovastatin (Fig. 2E and F). Obesity features excessive fat storage, which could be formed by the augmentation of adipocyte hyperplasia and hypertrophy. Therefore, we considered that the direct reason for weight loss was reduced fat mass. As the results showed, KA produced an obvious decrease in wet weights of WAT, while lovastatin did not show this effect (Fig. 2G). H&E staining revealed a significant decrease in the cross-sectional area and size of white adipocytes in DIO mice treated with KA compared to the vehicle group (Fig. 2H). In addition, KA or lovastatin treatment markedly reduced the remarkable accumulation of lipids in BAT (Fig. 2H). Moreover, KA or lovastatin treatment markedly reduced obesity-induced lipid accumulation (Fig. 2H). H&E staining showed that compared with DIO mice in the vehicle group, the damaged liver tissue was recovered in DIO mice treated with KA or lovastatin (Fig. 2H). The weight of the liver in KA or lovastatin-treated mice was also decreased (Fig. 2I). In line with the findings from oil red staining, the concentrations of TG and TC were notably decreased in DIO mice that received KA or lovastatin treatment (Fig. 2J and K). In addition, the lipid-lowering effect of KA was dose-dependent and stronger than that of lovastatin (Fig. 2J and K). The findings indicated that KA decreases weight gain and lipid levels in mice with diet-induced obesity.

### 3.3 KA reduces insulin resistance and enhances glucose tolerance in DIO mice

**Fig. 3:**
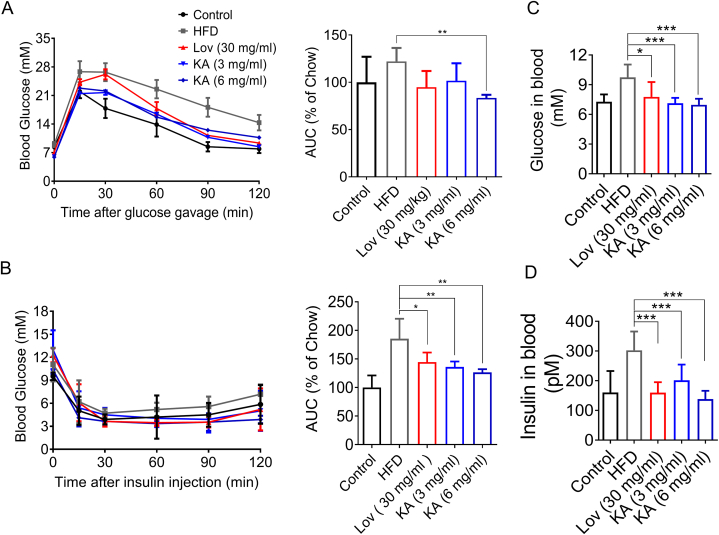
KA improves glucose intolerance and insulin resistance in DIO mice. (A) Effect of KA on glucose tolerance in DIO mice as determined by glucose tolerance test (GTT), and quantification of the area under the curve (AUC) from the GTT. (B) Effect of KA on insulin resistance in DIO mice determined by insulin tolerance test (ITT), and quantification of the AUC of the ITT. (C and D) Blood glucose (C) and blood insulin (D) in DIO mice were improved by KA treatment. Statistical analysis was done with one-way ANOVA (Dunnett's *post hoc* test). *p < 0.05, **p < 0.01, ***p < 0.001 *vs* Model.

Impaired fasting glucose or impaired glucose tolerance were considered common indicators of insulin resistance. In order to assess the impact of KA on insulin and glucose tolerance in DIO mice, both a glucose tolerance test (GTT) and an insulin tolerance test (ITT) were conducted on the mice. The results showed that impaired glucose and insulin tolerance were exhibited in DIO mice (Fig. 3A and B). Like lovastatin, KA significantly ameliorated glucose tolerance, with an approximate 25–30 % reduction in the area under this curve (AUC) of glucose levels in GTT (Fig. 3A). With regard to ITT, which was dramatically improved when KA was at the high dose, the area under this curve (AUC) of glucose levels in ITT was also conspicuously decreased (Fig. 3B). Furthermore, KA administration resulted in decreased fasting glucose and insulin levels (Fig. 3C and D). Together, although both lovastatin and KA could improve glucose tolerance, only KA treatment ameliorated insulin resistance and fasting glucose.

**Fig. 4:**
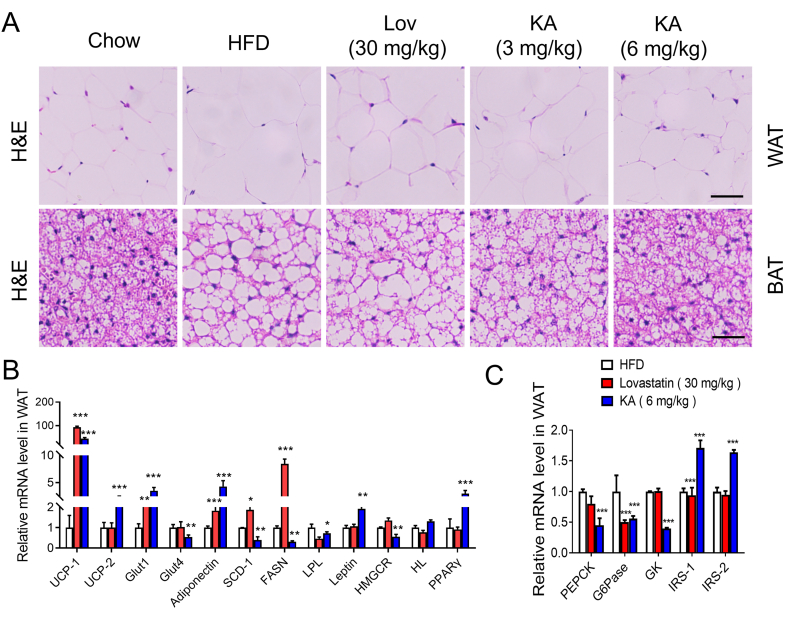
Effects of KA on lipid accumulation and metabolic gene expressions in adipose of DIO mice. (A) The Histological white adipose tissue (WAT) and brown adipose tissue (BAT). The WAT and BAT were stained with H&E. (B–C) Gene expression in WAT. Statistical analysis was done with one-way ANOVA (Dunnett's *post hoc* test). *p < 0.05, **p < 0.01, ***p < 0.001 *vs* Model.

DIO mice showed a significant decrease in adipocyte diameter in white adipose tissue (WAT) when treated with KA (Fig. 4A). Despite this, the adipose tissue size of mice treated with lovastatin did not significantly change (Fig. 4A). Adipose tissues in mice treated with lovastatin, however, did not show marked changes in size (Fig. 4B). To further assess the enhanced insulin sensitivity, we analyzed the gene expression of crucial metabolic genes in WAT. Our research found that KA treatment significantly reduced the gene expression of leptin in WAT, indicating that KA helped to normalize abnormal leptin levels. Similar to leptin, KA reduced the levels of glucose transporter type 4 (*Glut4*) expression (Fig. 4C). In WAT of KA and lovastatin-treated mice, gene expression analysis revealed the suppression of *Glut1*, *SCD-1*, *HMGCR*, *FASN*, *LPL*, and *UCP-2* (Fig. 4C), which are involved in lipid homeostasis. The expression of UCP-1 protein was notably higher in the WAT of mice treated with KA (Fig. 4C), which implied that KA might have the potential to promote adipose tissue browning, increase energy expenditure, and reduce body weight. KA seemingly boosted the expression of PPARγ in WAT. As shown in Fig. 4C, KA enhanced the expression of adiponectin. Additionally, the increased levels of adiponectin effectively suppressed the production of glucose in the liver by reducing the expression of genes involved in gluconeogenesis, such as glucose-6-phosphatase (*G6Pase*) and phosphoenolpyruvate carboxykinase (*PEPCK*), in the liver of overweight mice (Fig. 4C). Furthermore, KA decreased glycerol kinase (GK) expression and increased insulin receptor subtrate 1 (*IRS-1*) and *IRS-2* expressions in the WAT of DIO mice, suggesting that KA partially improved insulin resistance by repairing the impaired insulin signaling pathway caused by DIO (Fig. 4C). Collectively, these data demonstrate that KA improves lipid homeostasis, most likely through inhibiting DNL.

### 3.4 KA reduces the protein level of mSREBPs and relative target genes *in vitro*

**Fig. 5:**
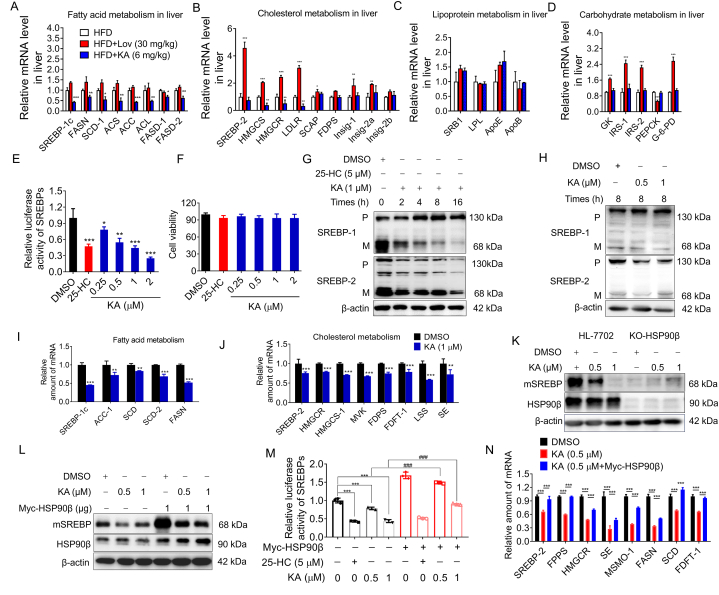
KA reduces the protein level of mSREBPs and relative target genes *in vivo* and *in vitro* (A–D) Indicated mRNA expression in the liver was detected by qRT-PCR. (E) The effect of KA on SREBP transcriptional activity was detected by reporter gene assay. (F) The effect of KA on cell viability was measured by MTT assay. (G) Effect of KA on protein level of SREBP in hepatocytes. (H) Effect of KA on SREBP protein expression in hepatocytes. (I) Effect of KA on gene expression related to fatty acid metabolism in hepatocytes. (J) Effect of KA on gene expression as related to cholesterol metabolism in hepatocytes. (K) KA does not reduce the level of SREBP in HSP90β KO HL-7702 cells. (L) The effect of KA on SREBP level was reversed by overexpressing HSP90β. (M) The effect of KA on SREBP transcriptional activity was reversed by overexpressing HSP90β. (N) The effect of KA on gene expression as related to fatty acid and cholesterol metabolism was reversed by overexpressing HSP90β. The uncropped images were provided in Supplementary Fig. 1. Statistical analysis was done with one-way ANOVA (Dunnett's *post hoc* test). *p < 0.05, **p < 0.01, ***p < 0.001 *vs* DMSO.

In order to further investigate how KA enhances lipid metabolism, we conducted additional analysis on the liver's lipid metabolism-related gene expression. It was demonstrated that KA did not impact the gene expression related to fatty acid oxidation and uptake (Fig. 5A–D). Nevertheless, KA notably decreased the hepatic expression of genes related to DNL, such as *HMGCS*, *HMGCR*, *LDLR*, *SS*, *FDPS*, *SCAP*, *Insig-2a*, *ACS*, *ATP-CL*, *SCD-1*, and *FASN*. It has been reported that HSP90β has the ability to control the transcriptional activity of SREBP, thereby regulating DNL in the liver. We hypothesized that KA can reduce hepatic DNL by inhibiting HSP90β and restricting SREBP transcriptional activity. KA was identified as an SREBP inhibitor by utilizing the human hepatocyte containing an SRE-driven luciferase. The well-known inhibitor of SREBP, 25-HC, demonstrated a significant decrease in luciferase activity in the positive control group (Fig. 5E). As shown in Fig. 5E, in a dose-dependent manner, KA greatly suppressed the activity of SREBP. Additionally, the viability of cells in the HL-7702 line was minimally affected by KA at a concentration of 2 μM, as indicated by the results of the cell viability tests. In line with the SREBP transactivation findings, KA had no effect on the precursor forms of SREBP-1 and SREBP-2 (Fig. 5G and H). Nevertheless, the expression of both endogenous mSREBP-1 and mSREBP-2 was notably reduced in HL-7702 cells exposed to KA, with KA's inhibitory impact being contingent on time and concentration (Fig. 5G and H). In HL-7702 cells, it was shown that a 1 μM concentration of KA significantly decreased the mRNA levels of various SREBP-1 target genes related to DNL, including *FAS*, *ACC1*, and *SCD-1*. SREBP-2 target genes including *SREBP-2*, *HMGCS*, *FDPS*, *HMGCR*, *MVK*, *LSS*, and *FDFT1* are involved in cholesterol synthesis (Fig. 5J). These data are consistent with the results in the liver *in vivo*. The above data indicated that the reduction of mSREBP by KA could potentially lead to a decrease in cholesterol and triglyceride accumulation *in vitro*. The findings align with the animal tests. To further demonstrate whether the regulation of SREBP transcriptional activity and hepatic DNL by KA depends on HSP90β, we first constructed HSP90β knockout hepatocytes (HL-7702/HSP90β KO). The depletion of HSP90β led to a significant decrease in mSREBP levels when compared to wild-type hepatocytes. Additionally, KA was unable to further decrease the amount of mSREBP-1c in HSP90β knockout cells (Fig. 5K). Following exogenous upregulation of HSP90β, there was a notable rise in the protein levels of mSREBP-1c in HL-7702 cells (Fig. 5L). The impact of KA on decreasing the mSREBP expression level was notably suppressed in hepatocytes with elevated HSP90β levels (Fig.5L). Significantly, the external rise in HSP90β in HL-7702 cells notably boosted the transcriptional activity of SREBP-1c, leading to an increase in the expression of downstream target genes like *ACC-1*, *FASN*, *SCD*, *FPPS*, *SE*, *HMGCR*, and *HMGCS* (Fig. 5M–N). Altogether, these results demonstrated that the regulation of SREBP transcriptional activity and hepatic DNL by KA depends on HSP90β.

### 3.5 KA promotes mSREBP degradation via the FBW7-mediated ubiquitin-proteasome pathway *in vitro*

**Fig. 6:**
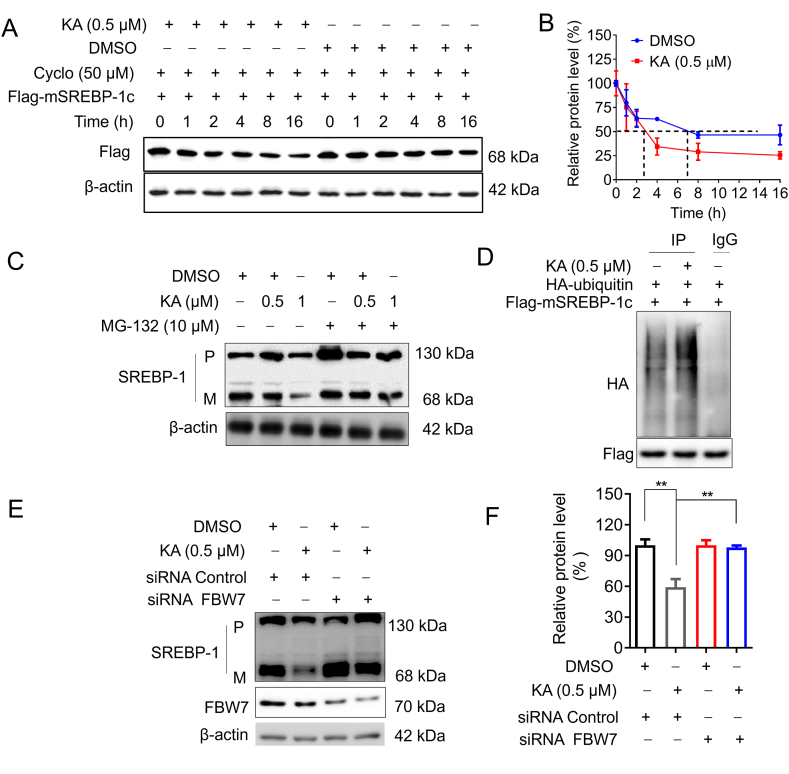
KA promotes mSREBPs degradation by FBW7-mediated ubiquitin-proteasome pathway (A) The degradation rate of mSREBPs in hepatocytes. (B) The signals detected on the membrane in (A). (C) the reduction of KA on SREBPs was reversed by MG-132. (D) The regulatory effect of KA on ubiquitination of SREBP-1. (E) The effect of KA on the SREBP level was reversed by knocking down FBW7. (F) The signals detected on the membrane in (E). The uncropped images were provided i n Supplementary Fig. 2. Statistical analyses were done with one-way ANOVA (Dunnett's *post hoc* test). *p < 0.05, **p < 0.01, ***p < 0.001 *vs* control siRNA or DMSO.

Reports indicated that HSP90β is responsible for preserving the stability of fully developed SREBP. Therefore, we speculated that KA promotes the degradation of mSREBP by the ubiquitin-proteasome. We first transfected HL-7702 cells with Flag-mSREBP-1c and then inhibited the transcriptional activity of cells with cycloheximide to investigate the degradation rate of mSREBPs. The results showed that KA could accelerate the degradation of mSREBPs (Fig. 6A and B). The impact of mSREBP depletion by KA was eliminated by MG-132, a proteasome inhibitor, indicating that the decrease in mSREBPs relied on the ubiquitin-proteasome pathway (Fig. 6C and D). Simultaneously, HL-7702 cells exhibited detectable ubiquitin levels of mSREBP-1c. As shown in Fig. 6E, the ubiquitin levels of mSREBP-1c were significantly increased in KA-treated hepatocytes. FBW7, as a component that recognizes substrates of E3 ubiquitin ligase, plays a role in breaking down m SREBPs. In HL-7702 cells exposed to KA, there was a clear reduction in the amount of mSREBP-1c, but this decrease was mitigated by the depletion of FBW7 (Fig. 6F). These results suggest that KA promotes ubiquitin-proteasome degradation by increasing ubiquitin levels of mSREBP, thereby inhibiting SREBP transcriptional activity.

### 3.6 KA promotes mSREBPs ubiquitination and proteasomal degradation through the Akt-GSK3β pathway *in vitro*

**Fig. 7:**
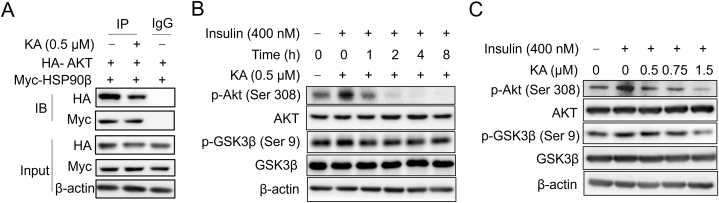
KA promotes mSREBPs ubiquitination and proteasomal degradation through the Akt-GSK3β pathway (A) Interaction of HSP90β and Akt was detected by WB. (B) Effect of KA on indicated protein levels in hepatocytes at different times. (C) Effect of KA on indicated protein level in hepatocytes. The uncropped images were provided in Supplementary Fig. 3.

We hypothesized that KA can inhibit the stability of mSREBP and hepatic DNL by inhibiting the interaction between HSP90β and Akt, and the GSK3β-FBW7 pathway. The study also found a direct interaction between HSP90β and Akt (Fig. 7A). The presence of KA in the cells resulted in the detachment of HSP90β from Akt, as shown in Fig. 7A, indicating that KA could potentially interfere with the HSP90-Akt interaction. KA significantly reduced Akt phosphorylation at Thr308 in a manner that depended on both the dose and time of exposure (Fig. 7B and C). Akt triggers the phosphorylation of GSK3β at Ser9, resulting in the deactivation of GSK function as shown in Fig. 7B and C. A summary of the modifications in detection parameters can be found in Table 2. Collectively, this information showed that KA, acting as a distinct blocker of HSP90β, enhances mSREBPs ubiquitination and proteasomal degradation via the Akt-GSK3β-FWB7 pathway.

**Table 2:** A summary of the change in detection parameters.

Figures	mediator	Detection index	Index change
Fig. 1A and B	*in vitro*	TC TG level	decrease
Fig. 1C–E	*in vitro*	neutral lipids and cholesterol level	decrease
Fig. 5E	*in vitro*	relative luciferase ability of SREBP	decrease
Fig. 5F	*in vitro*	cell viability of hepatocytes	No change
Fig. 5G and H	*in vitro*	SREBP protein level in hepatocytes	decrease
Fig. 5I and J	*in vitro*	mRNA level of lipid metabolism in hepatocytes	decrease
Fig. 6A and B	*in vitro*	The rate of KA degradation in hepatocytes	increase
Fig. 6C	*in vitro*	KA degrades SREBP through ubiquitin proteasome pathway	increase
Fig. 6D	*in vitro*	KA degradation of SREBP is dependent on E3 ubiquitin ligase FBW7	increase
Fig. 6D–F	*in vitro*	The FBW7-mediated SREBP degradation	increase
Fig. 7A	*in vitro*	Interaction of AKT with HSP90β	decrease
Figure B–C	*in vitro*	The level of *p*-AKT and *p*-GSK3β	decrease
Fig. 2A	*in vivo*	food intake	No change
Fig. 2B	*in vivo*	body weight	decrease
Fig. 2C–F	*in vivo*	TC/TG/LDL-C/HDL-C level in blood	decrease
Fig. 2G	*in vivo*	the weight of WAT	decrease
Fig. 2H	*in vivo*	Oil red staining of liver	decrease
Fig. 2, Fig. 4A	*in vivo*	H&E staining of liver/BAT/WAT	decrease
Fig. 2I	*in vivo*	the weight of liver	decrease
Fig. 2J and K	*in vivo*	TC/TG level in liver	decrease
Fig. 3A and B	*in vivo*	GTT/ITT	decrease
Fig. 3C	*in vivo*	Glucose in blood	decrease
Fig. 3D	*in vivo*	insulin in blood	decrease
Fig. 4B and C; 5A-D	*in vivo*	mRNA level of lipid metabolism in WAT/liver	decrease

## 4 Discussion

The epidemic of obesity has aroused widespread attention to find efficacious methods to control and cure it. It is urgent for us to find new drug treatments and drug targets in conflict with obesity and pertinent metabolic disorders. In the last ten years, HSP90 has become a major therapeutic target for cancer [23], neurodegenerative disorders [24], anti-viral [25], and *anti*-osteoclastogenesis [26]. Although there are promising opportunities in utilizing HSP90 inhibitors for treating illnesses, the role of four paralogs from the HSP90 chaperone family in diseases is still not well comprehended. In recent years, some reports have shown that alvespimycin (17-DMAG), a pan-HSP90 inhibitor 17-AAG derivate, decreases lipid content in mouse liver [27] and alleviates alcoholic liver injury [28]. In patients with NAFLD and obese mice, there was an increase in the expression of hepatic HSP90β instead of HSP90α. Hepatic HSP90β was also clinically relevant to serum lipid levels. Genetic manipulation or pharmacological methods have demonstrated that blocking HSP90β can greatly improve obesity-related fatty liver disease, type 2 diabetes, and atherosclerosis by suppressing SREBP function. KA is a diterpenoid with a unique structure that differs from all other natural product inhibitors of HSP90, including GA, radicicol, novobiocin, and purine-scaffold compounds [22]. Surprisingly, it was shown that KA forms a covalent bond with an unidentified cysteine 420 in the central region of HSP90, leading to impairment of their client kinases. The research revealed that KA has the ability to suppress hepatic DNL, lower blood lipid levels, prevent lipid accumulation in the liver and WAT, enhance insulin sensitivity, and reduce hepatic steatosis.

Despite advances in health care and medicine, an effective but safe approach to treat obesity has not yet been found. For example, statins exhibit good outcomes in treating hypercholesterolemia. However, rhabdomyolysis and renal toxicity remain major concerns in patients [29]. Over the past ten years, Brown and Goldstein's laboratory has found that SREBPs play a key role in controlling DNL [30]. Interestingly, despite insulin resistance, hepatic SREBPs are normally active in diet-induced and genetically obese mice [31], possibly due to ER stress [32]. These findings align with the evidence that DNL also rises in individuals with NAFLD and type 2 diabetes [33]. These findings offer the opportunity to develop anti-obese drugs with new strategies. After being activated by proteolysis, nuclear SREBPs combine signals from various pathways by undergoing posttranslational modifications such as phosphorylation, ubiquitination, and acetylation. Thus, multiple posttranslational processing steps of SREBPs could be targeted to their activities. For example, perturbations of ER to Golgi apparatus transport [34], and proteolysis and maturation of SREBPs by S1P and S2P [35], accelerate mSREBPs degradation and inhibit SREBPs transcription activity [35]. The research indicates that targeting SREBPs specifically could be a novel approach for developing medications to treat lipid disorders and type 2 diabetes. Nevertheless, compounds that block SREBP processing, like 25-HC, trigger LXR activation and ER stress, resulting in hepatic steatosis and insulin resistance [36]. KA suppressed SREBP activity via a different mechanism. By targeting HSP90β, it induces degradation of SREBP via the Akt-GSK3β-FBW7 pathway, while leaving LXR target genes and ER stress gene expression unaffected. The data indicates that KA has a unique edge when it comes to treating lipid disorders. Nevertheless, it is crucial to mention that we have not yet performed extended *in vivo* toxicological assessment, and the potential adverse effects need further investigation.

Here we first studied the cellular level, and the data showed that KA effectively decreased the levels of mSREBPs, which implied that KA could regulate lipid metabolism. KA prevented weight gain in obese mice by reducing lipid levels in a mouse model of obesity. Furthermore, KA rectified many issues related to obesity, enhancing hepatic steatosis and insulin sensitivity. Specifically, these biological changes depend on KA production and lead to the improvement of metabolic disorders caused by obesity. Hepatic steatosis and insulin resistance are severe complications related to obesity [37]. KA decreased lipid buildup in the liver of DIO mice without increasing microstructure. KA significantly inhibited the expression levels of SREBP targets, which were responsive to the decreased lipid contents. Furthermore, KA decreased the adipose tissue size and downregulated the expression of genes related to insulin resistance in obesity, including leptin, PPARγ, and adiponectin. Inadequate control of liver glucose production is a primary factor in glucose intolerance [38]. KA markedly reduced the levels of important genes involved in gluconeogenesis. Additionally, KA reduced the levels of GK while enhancing the levels of IRS-1 and IRS-2. The findings suggest that KA could enhance insulin sensitivity by upregulating gene expression, suppressing liver gluconeogenesis, and enhancing the insulin signaling pathway.

## 5 Conclusion

In conclusion, this study found that KA inhibited hepatic DNL and decreased the levels of mSREBPs *in vitro*. KA decreased serum lipids, restrained lipid accumulation in the liver and white adipose tissue (WAT), and improved insulin resistance, and hepatic steatosis. KA decreases the level of *p*-Akt ser308 by inhibiting the interaction between HSP90β and Akt. KA has the potential to be a key component in the pharmacological management of metabolic diseases caused by obesity.

## Ethics statement

This study was reviewed and approved by the Experimental Animal Ethics Committee of Fujian Medical University, ethics approval number: IACUCFJMU 2021-Y-1315.

## Availability of data and materials

All data related to the results of this study are available within the article.

## Data availability statement

Data included in article/supplementary material/referenced in article.

## CRediT authorship contribution statement

**Kun Miao:** Writing – review & editing, Writing – original draft, Visualization, Validation, Supervision, Software, Resources, Project administration, Methodology, Investigation, Formal analysis, Data curation, Conceptualization. **Yawei Zhao:** Writing – review & editing, Software, Investigation. **Ning Xue:** Writing – review & editing, Writing – original draft, Project administration, Investigation, Funding acquisition, Conceptualization.

## Declaration of competing interest

The authors declare that they have no known competing financial interests or personal relationships that could have appeared to influence the work reported in this paper.
