# Supplementary figures and images for: Gkongensin A, an HSP90β inhibitor, improves hyperlipidemia, hepatic steatosis, and insulin resistance
Source: Heliyon. 2024 Apr 9;10(8):e29367. doi: 10.1016/j.heliyon.2024.e29367 (PMC11036013; doi:10.1016/j.heliyon.2024.e29367)

**Supplementary Figure 1**


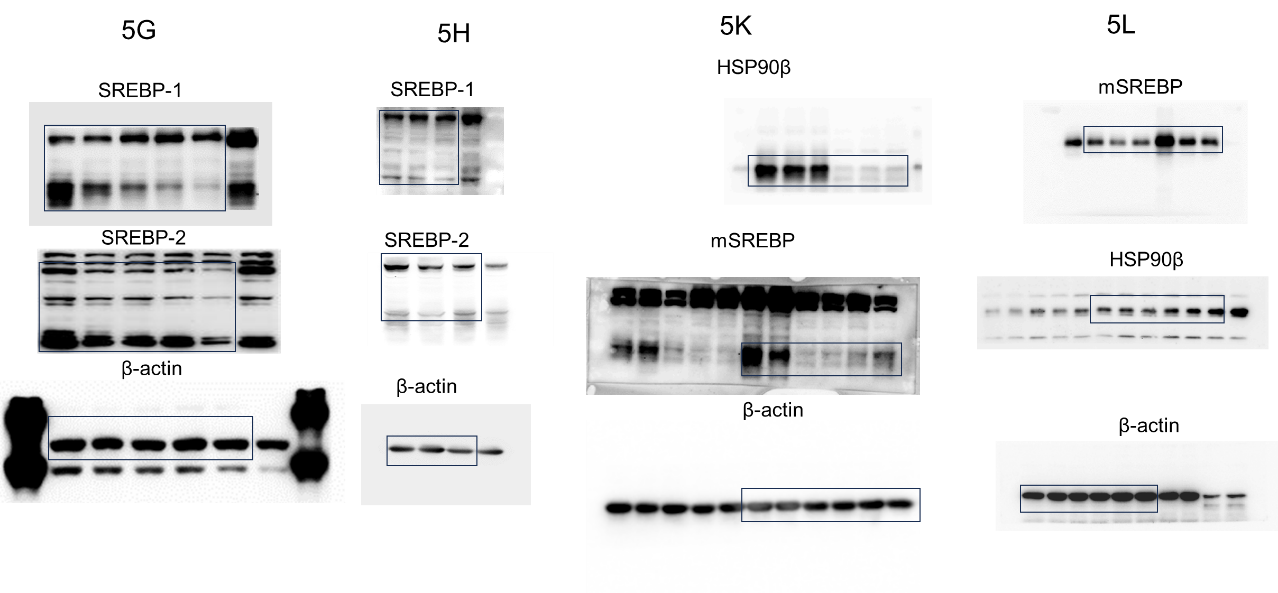


**Supplementary Figure 2**


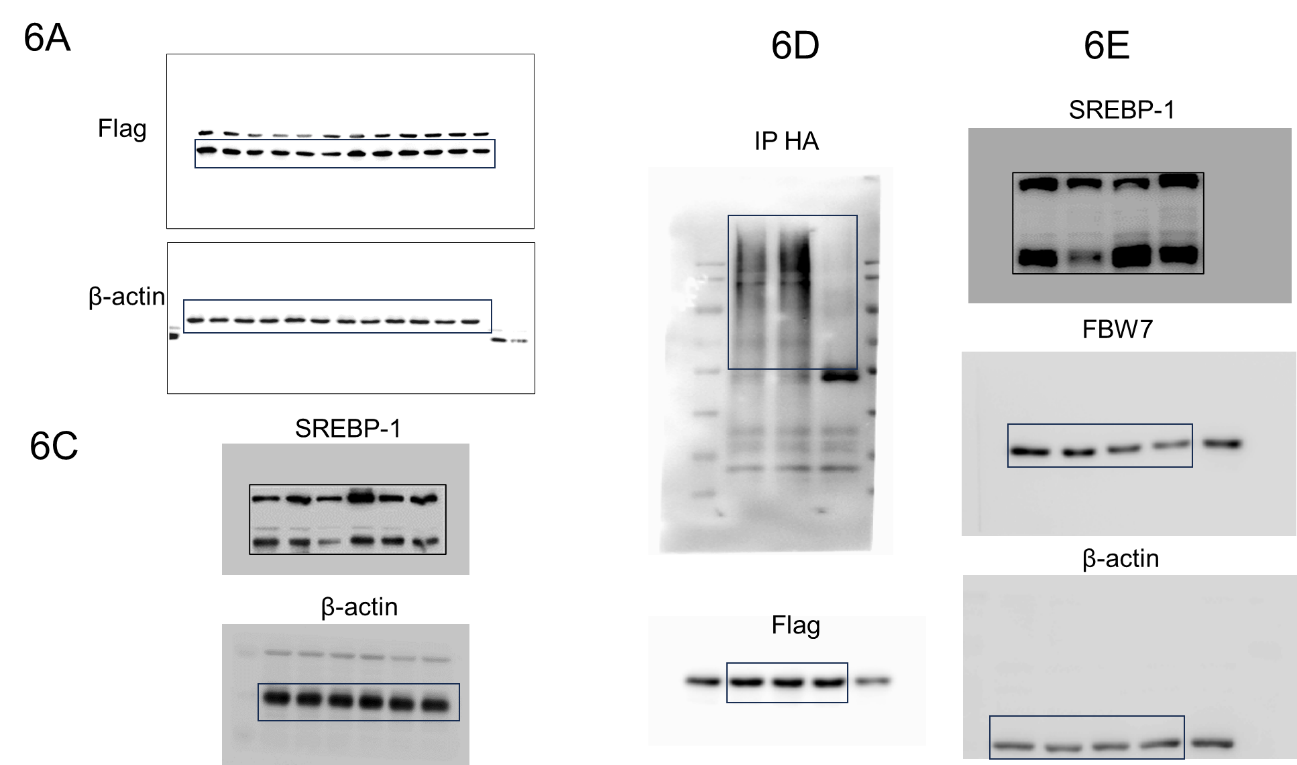


**Supplementary Figure 3**


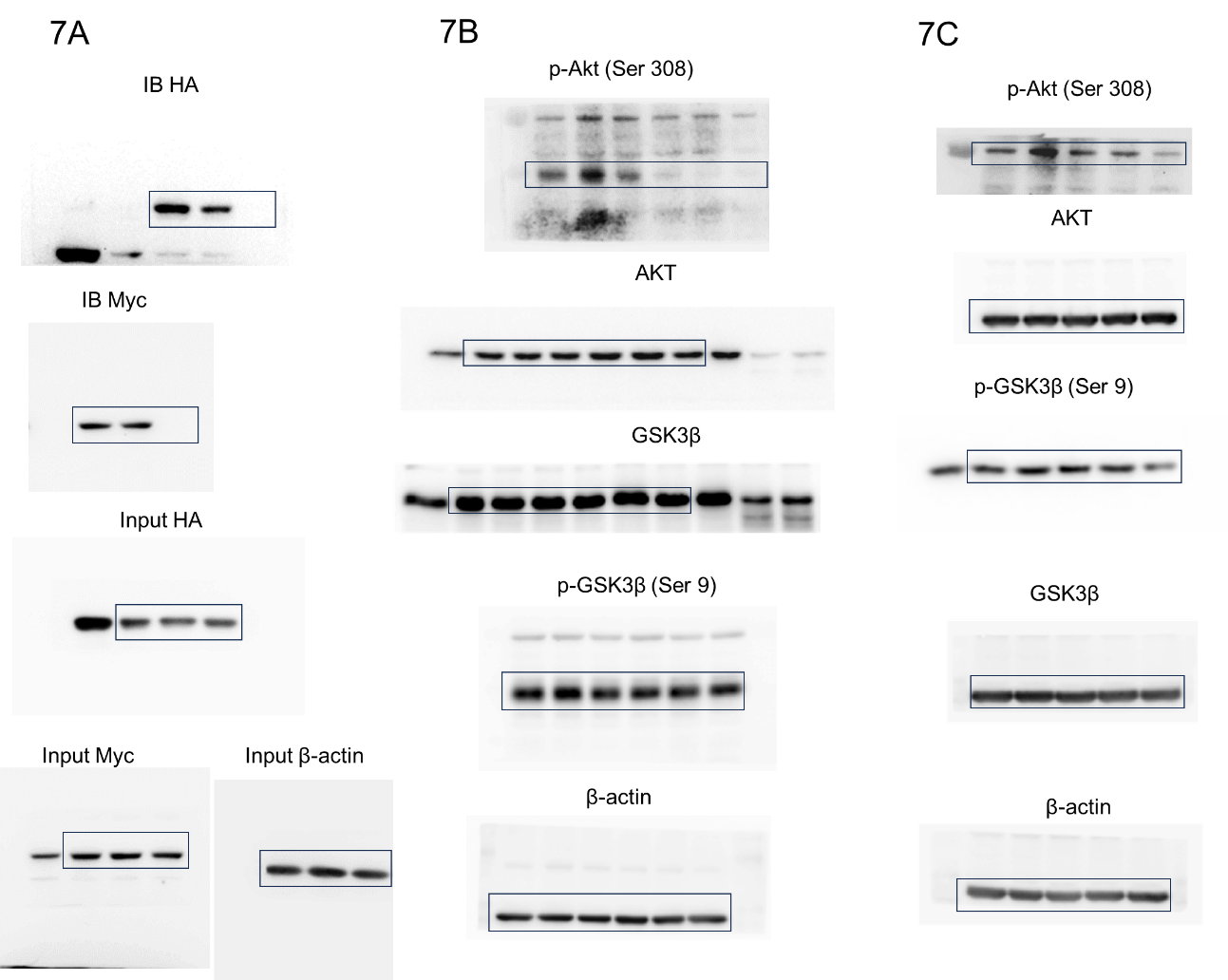

Supplement: Multimedia component 1 [file mmc1.docx]
